# Safety and physiologic effects of intranasal midazolam and nitrous oxide inhalation based sedation in children visiting Saveetha Dental College and Hospitals, India

**DOI:** 10.6026/97320630018026

**Published:** 2022-01-31

**Authors:** Neethu Ann Preethy, Sujatha Somasundaram

**Affiliations:** 1 Saveetha Dental College,Saveetha Institute Of Medical and Technical Sciences,Chennai,Tamil Nadu,India

**Keywords:** midazolam, nitrous oxide, conscious sedation, safety, children

## Abstract

A large number of patients avoid dental care due to anxiety. Various techniques are available for behaviour related management. Therefore, safety and physiologic effects of intranasal midazolam and nitrous oxide inhalation based sedation in children aged 4 to 8 years visiting Saveetha Dental College and Hospitals, India is of interest. 35 anxious patients aged 4 to 8 years were included in the study. The patient received either intranasal midazolam/nitrous oxide in the first visit and vice versa at the second visit. The onset of sedation, recovery time and procedure duration were recorded using a timer. Physiological parameters were recorded using a monitor. Safety scale was used for assessing prevalence of adverse reactions. There was no significant difference between the groups in safety scale scores, recovery time and procedure duration. Midazolam group showed a statistically significant faster onset of sedation and a statistically significant increase in heart rate at four recorded time-points. All the vitals were within the physiological limits. Thus, intranasal midazolamis a safe alternative to nitrous-oxide sedation in completing the intended dental treatment while managing the anxious children in dental clinic.

## Background:

Dental fear and anxiety prevail to be a major obstacle for paediatric dentists in rendering successful treatment to children as it impedes, or even precludes the quality of dental care to be provided [1,2]. Dental anxiety denotes a state of apprehension that something dreadful is going to happen in relation to dental treatment, and is coupled with the sense of losing control [3]. It was observed in a study that 60% of the children who were experiencing dental fear presented with behaviour management problems, out of which 25% of children were experiencing anxiousness [4].

Behaviour management serves to be that one aspect and the cornerstone factor that sets apart paediatric dentistry from all other dental specialities [5]. Pharmacological or advanced behaviour management techniques should be considered in cases where the non-pharmacological or psychological behaviour management techniques prove to be unproductive [6]. The main advantage of using pharmacological behaviour management is the decreased interruption of the dental treatment exhibited by the child experiencing behaviour management problems [7].Several factors play a role in the decision upon the type of pharmacological behaviour management to be provided such as age of the patient, pre-operative anxiety, extent of patient's dental needs, risk involved with pharmacological management, safety, parental expectation and cost [8]. AAPD has formulated goals and given guidelines for using basic and advanced techniques in managing paediatric dental patients [9-10].

Sedation was originally discussed under conscious and deep sedation [11]. However, the modern day concept has modified the broad term 'conscious sedation' to minimal sedation previously called 'anxiolysis' and 'moderate sedation' previously called 'conscious sedation'. [12] The American College of Emergency physicians (ACEP) has given the terminology of 'procedural sedation' which is defined as 'a technique of administering sedatives or dissociative agents with or without analgesics to induce a state that allows the patient to tolerate unpleasant procedures while maintaining cardio-respiratory functions. It is intended to result in a depressed level of consciousness that allows the patient to maintain oxygenation and airway control independently [12].

Oral route leads to a slow onset of action and also a longer recovery period [13]. Rectal route is considered to be safe, painless and also reliable for younger children, but it might be embarrassing for adolescents and dental staff [14]. Intravenous route and intramuscular route have a major advantage of titration of drugs but the inherent fear of needles in paediatric patients makes the administration quite difficult [15]. The technique of intranasal drug administration requires minimum co-operation of the patient and thus has gained interest in the field of Paediatric Dentistry as the drug is absorbed directly into the systemic circulation due to the highly vascular nasal mucosa and also there is no strict sterile technique for the administration of the drug [16].

Ketamine, midazolam, dexmedetomidine and sufentanil are commonly used drugs administered through the intranasal route [17-18]. Among them, midazolam- a newer generation benzodiazepine has been mentioned as "potentially the ideal sedative agent" [19] for its wide toxic/therapeutic ratio and safety margin [6]. Hence it has been chosen over other agents for sedation in the present study. Midazolam has a relatively short half-life and thus has a rapid onset and recovery [20]. It can be administered orally, intranasally, sublingually, rectally or intravenously and has a rapid elimination half-life, produces anterograde amnesia, is a muscle relaxant and also yields no active metabolites [21-22]. Midazolam when administered through the intranasal route has a faster onset of action since it avoids the hepatic "first pass metabolism" and gets absorbed through the cribriform plate into the brain resulting in an increased bioavailability level [23,24]. It exerts its sedative, hypnotic, anxiolytic and anterograde amnesia effects by action on GABA associated benzodiazepine receptors [25]. Thus, due to the above mentioned advantages, atomized administration of intranasal midazolam was used in the present study.

Nitrous oxide- oxygen sedation has been considered to be the standard sedative technique by the Council of European Dentists. Nitrous oxide is a colourless, sweet-smelling gas and exerts its analgesic and anxiolytic properties by causing depression of the central nervous system [26]. This route offers the advantage of titration of the dosage and has rapid induction and recovery while displaying absence of any systemic hazards. However, the patient's acceptability of the mask confers to be a major factor affecting its use. Thus it's a very technique sensitive procedure and requires continuous induction of the agent throughout the treatment which might pose a difficulty of its use in very fearful children [27]. Therefore, it is of interest to compare the effect of atomized intranasal midazolam (0.3 mg/kg body weight) with nitrous oxide oxygen sedation in the evaluation of safety, physiological effects, and the onset and level of sedation of the drugs.

## Materials and methods:

### Study design:

The present study is a randomized split mouth crossover clinical trial conducted in the Department of Paediatric and Preventive Dentistry, Saveetha Dental College, Chennai,in accordance with the guidelines given by the CONSORT checklist. The study design was reviewed and approved by the Institutional Review Board (SRB/SDMDS07/18PEDO/24) and registered in CTRI prospectively (CTRI/2019/09/021381).

### Inclusion criteria:

1) Children requiring pulpectomy treatment in the lower two quadrants with the use of inferior alveolar nerve block.

2) Children in the age group of 4-8 years.

3) Children having physical status of ASA type 1.

4) Children exhibiting negative and definitely negative behaviour rating.

#### Exclusion criteria:

1) Children with cognitive impairment

2) Children with any respiratory condition that makes breathing difficult through the nose.

3) Children with any history of systemic illness and patients who require special physical and mental requirements.

4) Children who have recently used medication such as erythromycin or anticonvulsants that interfere with the pharmacokinetics of midazolam.

5) Children with known hypersensitivity to benzodiazepines.

#### Sample Size Calculation:

The sample size of the present study was determined from a prior pilot study following a similar study design using G-power with 95% power and α error to be 0.05. The sample size was estimated to be 28 per group. The sample size was increased by 25% to arrive at a total sample size of 35 patients taking into account the possibility of any drop-outs or discontinued intervention in the study group. Thus, a total of 35 anxious paediatric patients aged 4-7 years requiring bilateral pulpectomy that required administration of inferior alveolar nerve block were assessed for eligibility for the study.

#### Randomization and Allocation concealment:

**Figure 1:**
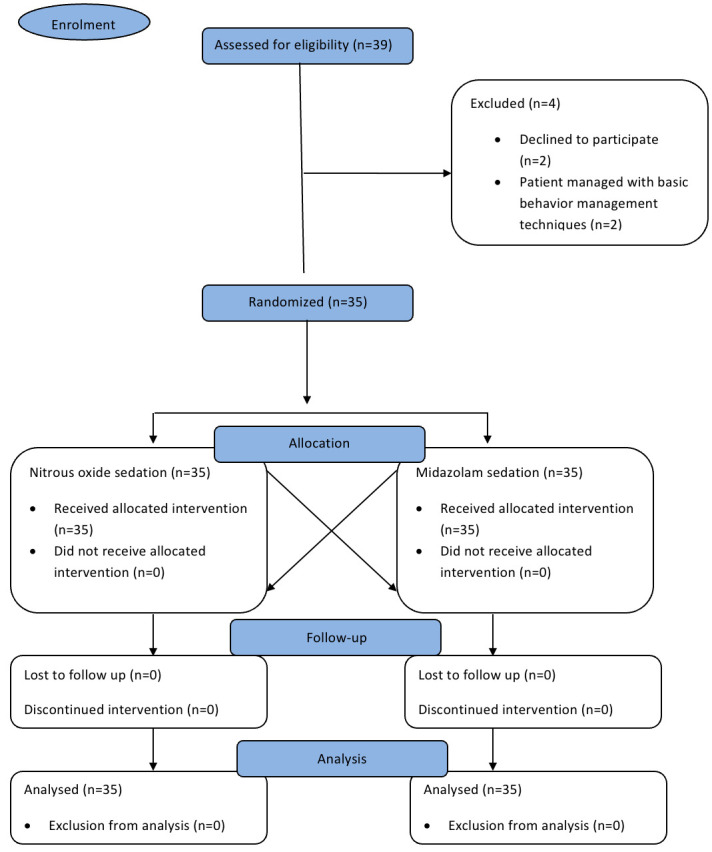
Methodology flowchart

The participants included in the study were randomized using block randomization. Research randomizer software was used by a postgraduate student to generate a sequence for a block of 35 with either 1 or 2 treatment protocol for the first appointment (where, 1=intranasal midazolam group and 2=nitrous-oxide oxygen group). Similarly, a separate randomization sequence using the same software was done for the site of treatment to be performed. In the second appointment, the other intervention was used for pulpectomy on the contra-lateral side (Figure 1). Sequentially numbered envelopes were used for concealment of the sequences by the post-graduate student which was then opened by the operator at the time of dental treatment and the allocated intervention protocol was followed. It was a single blinded study in which the patients were blinded but the operator as well as the evaluator could not be blinded since the two methods of induction were easily distinguishable.

#### Study procedure:

The study protocol, risk and benefits of the treatment were explained to all the parents/guardians and a written informed consent was obtained before inclusion of the child in the study. Parents who did not give consent were excluded from the study and an alternative treatment protocol was provided to them. The behaviour of all the patients were assessed prior to inclusion in the study based on the Frankl's behaviour rating scale. Prior to the inclusion of a child into the study, basic behaviour modification was attempted. Only those patients on whom basic behaviour management failed were included. A comprehensive general health evaluation was done by the Professor of the Department of Anaesthesiology, Saveetha Medical College, Chennai (India) for all the patients prior to the enrolment. This evaluation included tonsils and adenoid assessment, mouth-breathing, speech, hypo-nasality, snoring, airway and chest examination. The parents were explained about the fasting protocol in accordance with the American Academy of Paediatric Dentistry guidelines [9] and were asked to ensure the child followed it prior to the treatment. The parents were instructed not to feed any solid or non-clear liquid for 4 hours before the sedation procedure. All the instructions were explained in verbal as well as in written formats to the parents. On the day of sedation, each patient was re-examined for physical fitness by the Professor of Anaesthesiology. The patient's body weight was measured using a weighing scale and noted. The physiological parameters such as oxygen saturation, heart rate, and respiratory rate, systolic and diastolic blood pressure were monitored and recorded throughout the procedure till discharge. The operator performing all the dental procedures received special training to administer nitrous-oxide oxygen and intranasal midazolam sedation. All the patients were continuously monitored by the anaesthetist throughout the procedure.

#### Intranasal Midazolam Administration:

**Figure 2:**
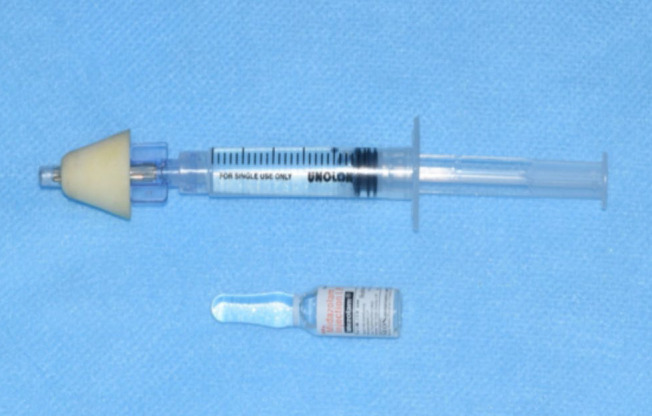
Mucosal atomization device attached to syringe and 5mg/ml midazolam vial

Intranasal dose of 0.3mg/kg weight of midazolam hydrochloride (trade name Mezolam 5mg/ml, Neon Laboratories Ltd) was administered for every patient using mucosal atomization device (Wolf Tory Medical, Salt lake city, Utah) attached to a 2ml syringe (Figure 2). A concentrated dosage of midazolam delivering 5mg per ml was used in this study to minimize the volume of the drug administered to the patient. The precise dose was calculated according to the weight of the child and in case of decimals, the dose was rounded off to 0.5mg dose more than the calculated value. After the establishment of the vital baseline values, the patient was explained the entire procedure for the administration of the drug through the nose using euphemism. "Magic spray that puts you to sleep" was the euphemism used. A bolus dose of 0.3mg/kg was administered to each patient and the patient was observed and signs and onset of sedation were monitored. The level of sedation was also noted down at baseline, after 5 minutes, 15 minutes and at the end of the procedure according to the Ramsay sedation scale (Table 1 - see PDF).The administration of local anaesthesia was initiated after the patient appeared, relaxed with slurring or slowing of speech. The physiological parameters were monitored and recorded at different stages of the procedure: at baseline, during administration of local anaesthesia, during pulp therapy procedure and at the end of the procedure.

#### Nitrous oxide-oxygen administration:

**Figure 3:**
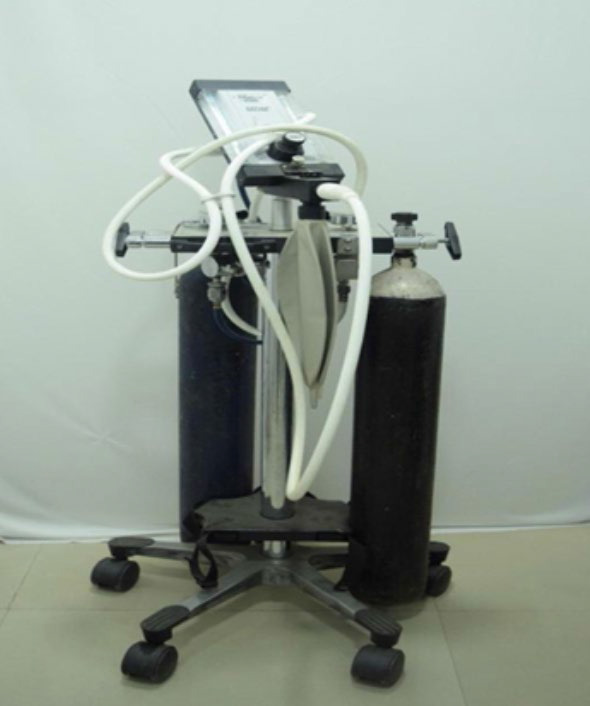
Matrx Porter Digital Relative analgesia machine

**Figure 4:**
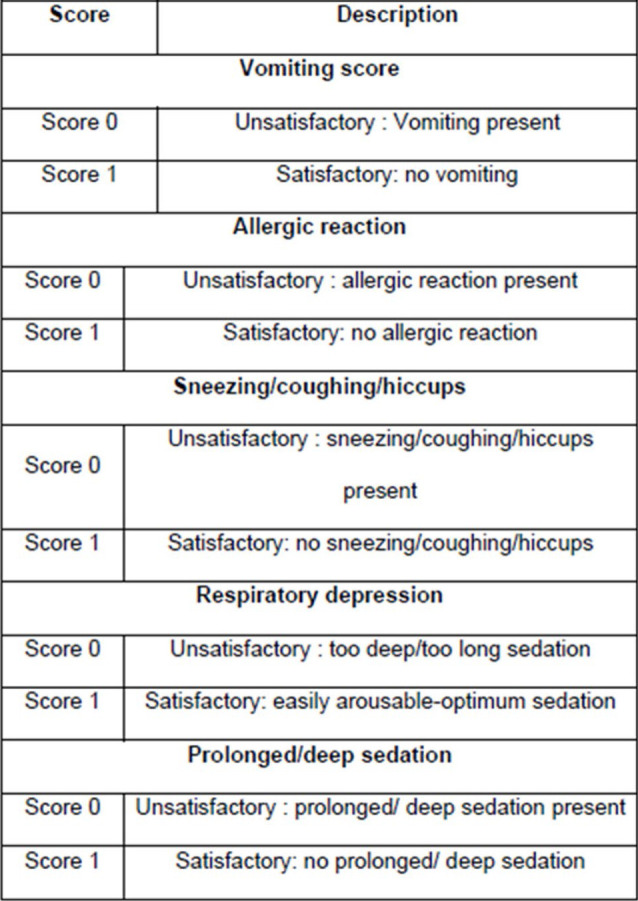
Safety Scale

Nitrous oxide-oxygen was administered using Matrx Porter Digital Relative analgesia machine in the concentration of 30%-70% nitrous oxide-oxygen (Figure 3). The placement of the nasal mask was manually explained to every child using the tell show do method and astronaut's mask was used as a euphemism for the placement of the nasal mask. The baseline vitals were established prior to induction. Initial administration of 100% oxygen for 2-3 minutes was initiated to determine the flow rate. A pre-adjusted mixture of nitrous oxide- oxygen was administered and maintained throughout the procedure. The onset of sedation and onset of satisfactory sedation was observed and recorded. The administration of local anaesthesia was initiated after the first signs of sedation such as relaxed appearance and slurred speech. At the termination of the procedure, 100% oxygen was administered for 5 minutes. The level of sedation was noted according to the Ramsay sedation scale (Table 1 - see PDF) at baseline, after 5 minutes, after 15 minutes and at the end of the procedure after administering 100% oxygen for 5 minutes. Similarly, the physiological parameters were monitored and recorded at different stages of the procedure: at baseline, during administration of local anaesthesia, during pulp therapy procedure and at the end of the procedure. All the variables of the study were evaluated by a separate observer present during the entire dental procedure. As the two methods of induction were easily distinguished, the operator as well as the evaluator could not be blinded. Any adverse reaction which occurred such as vomiting, allergic reactions, coughing, sneezing, hiccups and any prolonged or deep sedation caused by either of the drugs was recorded using a scale given by Shashikiran et al. in 2006 [28] (Figure 4). After the end of the dental procedure, the patient was shifted and monitored in the recovery room. The patient was discharged when the recommended discharge criteria given by AAPD were met [29]. All the patients were followed up till the next day through phone calls to evaluate if there were any adverse reactions that occurred in the post-treatment period.

#### Statistical Analysis:

All the acquired data was entered in the spreadsheet and the analysis was done using SPSS software version 23 (IBM SPSS Statistics for Windows, Version 23.0, Armonk, NY: IBM Corp. Released 2015). Independent sample t-test was used to compare the scores on the same variable. To compare proportions between groups, Chi-Square test was applied.

### Results:

A total of 35 participants were recruited for the trial with a mean age of 5.66 ± 0.77 years. Among them, 51.4% (n=18) of the participants were males and 48.6% (n=17) were females. All the 35 patients completed the planned treatment under the assigned sedation technique. The onset of sedation and satisfactory sedation were recorded and compared between the two groups. The difference was found to be significant with a lesser time required for the onset of sedation (p=0.000) and onset of satisfactory sedation (p=0.000) with the midazolam group compared to nitrous oxide group (Table 2 - see PDF).

The physiological parameters such as heart rate, respiratory rate and oxygen saturation, systolic and diastolic blood pressure were measured at five different time points (Table 3 - see PDF). All vital signs remained within acceptable clinical limits with both types of sedation used in the study. Independent-test was used to compare the physiologic parameters between two groups. There was a statistically significant increase in the heart rate during local anaesthetic administration, at maximum sedation, during pulp therapy procedure and at the end of the dental procedure in the midazolam group when compared to the nitrous oxide group (p=0.00).

**Figure 5:**
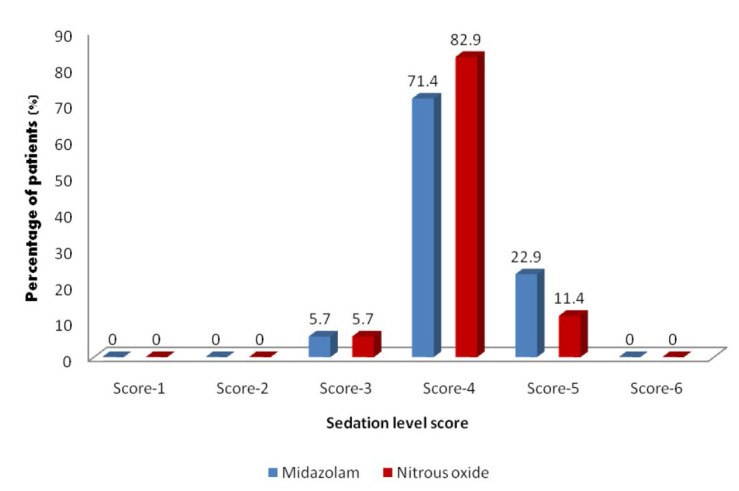
Distribution of patients according to the Ramsay scale sedation score at maximum sedation. Distribution of patients according to the Modified Ramsay scale sedation score at maximum sedation shows that the level of sedation was observed to be in the range of scores (scores 3-5) signifying moderate sedation in both the groups.

**Figure 6:**
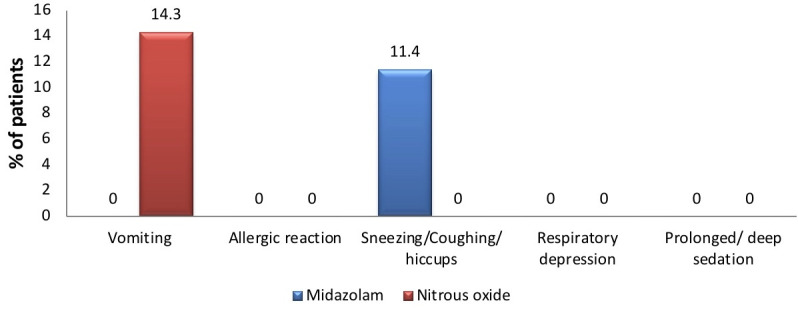
Graph showing prevalence of reactions using safety scale. According to the safety scale scores, 14.3 % participants displayed vomiting during nitrous oxide sedation. And, 11.4% participants showed sneezing/coughing/hiccups during midazolam sedation. No other adverse effect was reported during sedation in either of the groups.

**Figure 7:**
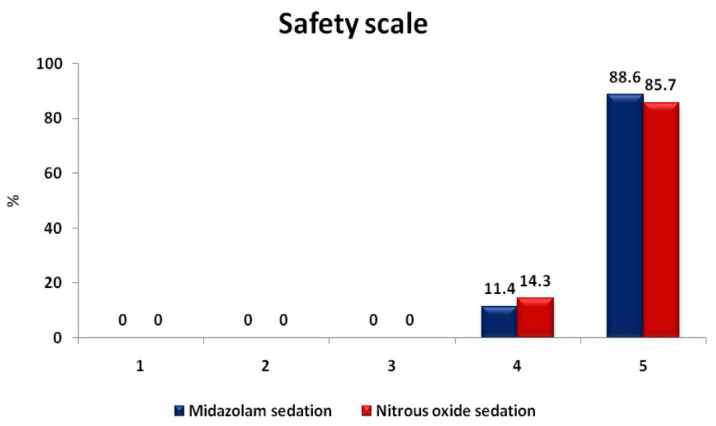
Distribution of patients according to the total score in Safety scale. The safety scale scores show that 88.6% of midazolam sedation group and 85.7% of nitrous oxide sedation group displayed a score of 5 whereas 11.4% of midazolam sedation group and 14.3 % of nitrous oxide sedation group displayed a score of 4.

Chi-square test was used to measure the level of sedation according to the Modified Ramsay sedation scale. The level of sedation was observed to be in the range of scores signifying moderate sedation in both the groups with no statistically significant difference.(Table 4 (see - PDF, Figure 5) Independent samples t-test was used for comparing the duration of the procedure and recovery time between the groups, and there was no statistically significant difference found between the two groups (Table 5 - see PDF). Chi-square test was used to compare the safety scale scores, where 5 participants displayed vomiting during nitrous oxide sedation. And, 4 participants showed sneezing/coughing/hiccups during midazolam sedation. No other adverse effect was reported during sedation in either of the groups (Table 6 - see PDF, Figure 6,Figure 7).

## Discussion:

Pharmacological behaviour management is considered to be an effective alternative in children who are anxious or display uncooperative behaviour and in whom the basic behaviour management strategies fail to produce the desired effect [30]. Among the various pharmacological techniques practised in pediatric dentistry, conscious sedation is gaining popularity considering the complication and increasing cost of general anaesthesia treatment [31]. Thus in the present study, two methods of delivering conscious sedation, that is intranasal midazolam and nitrous oxide-oxygen sedation are compared. A split mouth design was used in the present study. This was advocated to use both types of conscious sedation in all patients included in the study. Thus, it would result in less variation in the assessed outcome after sedation. The present study uses 0.3mg/kg of midazolam via intranasal route of administration. Although oral route is considered to be the most common route of drug administration, the present study uses intranasal route due to the bitter taste of the drug that cannot be masked easily in oral route [18] thus making inadequate quantity of the drug to be ingested providing a variable sedative effect.

We used mucosal atomization devices for midazolam administration which produces a fine 30µ-m particle spray that increases the area of absorption of drugs. Also, the semi-permeable soft plug in the mucosal atomization device cushions the naris thereby preventing the back-leak of the drug. Thus, it provides rapid absorption of the drug into the systemic circulation [32]. In a previous study by F. Gilchrist et al, intranasal midazolam was used in the dosage of 0.25mg/kg which provided adequate anxiolysis to complete the intended dental procedure [33]. Another study by Fuks et al. [34] revealed that 0.2mg/kg midazolam was observed to have similar effectiveness as 0.3mg/kg drug. However, in the study midazolam was used in combination with 50% nitrous oxide sedation. But, as the present study did not use midazolam in combination with other agents, higher dosage of 0.3mg/kg of midazolam was used for achieving conscious sedation. A previous study by Bahetwar et al. also used 0.3 mg/kg of midazolam without combining with other agents for achieving sedation [17]. Nitrous oxide was used in the present study at a concentration of 30%-70% delivered as a premixed dose. A concentration of 30% nitrous oxide was used since previous studies have demonstrated that 20%-30% concentration provides adequate sedation without the risk of over sedating the child [35]. A pre-mixed dose was given to standardize the dose of nitrous oxide administered for all patients included in the study undergoing nitrous oxide sedation. Dental treatment was successfully completed in both the methods of sedation with good overall behaviour.

Intranasal midazolam has shown to have a faster onset of sedation compared to nitrous oxide sedation. No other studies have evaluated the onset of sedation or satisfactory sedation comparing intranasal midazolam and nitrous oxide sedation. The level of sedation was measured according to Modified Ramsay sedation score at different time periods during the procedure in both the groups. It was observed that intranasal midazolam was as effective as nitrous oxide sedation to achieve an adequate level of sedation and complete dental treatment successfully. We show that the physiologic parameters that include systolic and diastolic blood pressure, oxygen saturation, heart rate and respiratory rate pre-operatively, during the administration of local anaesthesia, at maximum sedation, during pulp therapy procedure and at the end of the procedure. There was an increase in the heart rate observed in the midazolam group during administration of local anaesthesia, at maximum sedation, during pulp therapy procedure and at the end of the procedure. This may be due to the effect of adrenaline delivered during local anaesthesia administration combined with the predominance of sympathetic activity of midazolam on heart rate.

Other studies have reported an increase in the heart rate after administration of local anaesthetic agents with adrenaline which is in accordance with the results of the present study. However, these studies did not compare heart rate between two sedative agents [36,37]. The heart rate was observed to be significantly higher in the midazolam group in the present study. No other study has compared heart rate or other physiological parameters between intranasal midazolam and nitrous oxide sedation in children. For any drug to be deemed appropriate for patient use, a satisfactory safety profile is of foremost importance. The main objection associated with the use of midazolam for conscious sedation is the risk of occurrence of paradoxical reactions. These reactions are rare and are more commonly associated with oral route in children [38,39]. However, there was no incidence of paradoxical reactions associated with intranasal midazolam in the present study. The most common complications reported in other studies with use of intranasal midazolam were coughing and sneezing. It may be due to the increased volume of the drug trickling through the oropharynx [33]. Thus, in the present study, the concentration of the drug used for intranasal administration of drug was 5mg/ml delivered by mucosal atomization device. A highly concentrated drug was used to minimize the volume of drug to be administered thereby preventing any adverse effects. 11% of the patients in the present study reported an incidence of sneezing with use of intranasal midazolam. However, there was no burning sensation observed in this study. Another study which used a highly concentrated drug of intranasal midazolam demonstrated similar results to that of the present study [17].

The most common complication reported with nitrous oxide sedation was vomiting in 14% of the patients. A low incidence of vomiting was reported because of strict pre-operative fasting followed. Nevertheless, this complication did not affect the overall treatment outcome or completion of the procedure. Another study reported a 2.2% incidence of vomiting with the use of nitrous oxide sedation [40]. A study by Musani and Chandan involving the use of nitrous oxide sedation did not report any incidence of vomiting [5]. No other adverse effects were observed in the present study due the moderate dose of nitrous oxide sedation used. In accordance with physiological parameters and reported side effects, both the drugs used in the study showed accepted profiles. The present study does not evaluate the behaviour outcome associated with the sedation and the preference of the technique used in children. These factors would further impart a better comparison for the two different methods of sedation used in the study for dental treatment of pediatric patients. In addition, this study only evaluated two most commonly used agents for conscious sedation in children requiring pulpectomy. Thus, comparison of multiple drug regimens used for conscious sedation would assist in determining the better method of conscious sedation in pediatric patients undergoing dental treatment.

## Conclusion:

Results show that 0.3 mg/kg intranasal midazolam is as plausible as 30% nitrous oxide in providing a safe and satisfactory sedation for carrying out pulpectomy treatment in pediatric dental patients. Intranasal midazolam showed a faster onset of sedation as well as satisfactory sedation when compared to nitrous oxide sedation. Sedation of a child requires high knowledge and skill of the particular technique chosen. Therefore, it is crucial that any clinician who undertakes such treatment is completely proficient to do so.

## Ethical approval:

The ethical aspect and study design was reviewed and approved by the Institutional Review Board of Saveetha Institute of Medical and Technical Sciences, Chennai, India.
